# Perception of artificial intelligence and machine learning applications in the Nigerian healthcare sector: A cross-sectional study

**DOI:** 10.1371/journal.pgph.0006124

**Published:** 2026-05-28

**Authors:** Obi Peter Adigwe, Godspower Onavbavba

**Affiliations:** National Institute for Pharmaceutical Research and Development, Abuja, Nigeria; Universitas Muhammadiyah Aceh, INDONESIA

## Abstract

Artificial intelligence (AI) has significant benefits across various facets of healthcare delivery, notably in personalised pharmacotherapy and the development of targeted health interventions for specific demographic groups. This study aimed to comprehensively evaluate the applications of AI and machine learning (ML) from the perspectives of healthcare professionals in Nigeria**.** A cross-sectional study design was adopted, and responses were obtained from healthcare professionals across the six geopolitical zones in Nigeria. Data were collected with the aid of a well-structured questionnaire, and a stratified multistage sampling method was employed. Statistical Package for Social Sciences (SPSS) software version 25 was used to analyse the data generated. The results of descriptive analysis and inferential analysis were generated and discussed appropriately. Pharmacists (27.7%), physicians (24.5%), and nurses (19.3%) comprised the largest professional groups, with females representing 55.7% of participants. Most respondents (68.3%) were aged 18–30 years. Two-thirds (67.3%) agreed that ML algorithms can enhance research processes, and a similar proportion (69.5%) believed AI could accelerate drug discovery. Regarding clinical decision-making, 66.6% indicated that AI can support physicians by processing unstructured data, while 77% felt AI and ML could improve pharmaceutical manufacturing. This study revealed positive perceptions from healthcare professionals regarding the applications of AI and ML in the Nigerian health sector. The findings from this study indicate the readiness of the health workforce for a large-scale implementation of AI technologies in the sector.

## Background

The healthcare sector is under immense pressure due to rising morbidity rates and the need to customise clinical interventions based on individual medical requirements [1]. Additionally, the Nigerian healthcare system faces various challenges, including rapid population growth, shortage of healthcare professionals, and limited healthcare infrastructure [2]. Artificial intelligence (AI) has the potential to address several of these challenges due to its precision capabilities [3]. Machine learning (ML) algorithms can analyse large volumes of patient data, including genomics, medical records, imaging data, and lifestyle factors, to identify patterns, predict health outcomes, and recommend targeted interventions [4]. Similarly, a shortage of the health workforce can be alleviated through the optimisation of medical workflow, thereby reducing the burden on healthcare professionals. This ensures the productive use of human resources and better health outcomes [5]. Furthermore, AI-powered telemedicine platforms can provide remote consultations and diagnoses, thereby increasing the reach of healthcare services to rural areas [6].

In the past, the concept of AI in healthcare was often associated solely with robots performing surgical procedures. However, the field has evolved rapidly, leading to a broader understanding of its applications. The emergent ecosystem has now been categoried into groups that include physical AI and virtual AI [7]. Physical AI encompasses intelligent medical devices, wearables, and surgical robots [8]. On the other hand, virtual AI refers to software-based applications and algorithms that analyse complex medical data to provide insights, predictions, and clinical decision-making support [9].

The main subsets of virtual AI include ML, deep learning, natural language processing, image recognition, and expert systems [10]. ML is an AI algorithm that enables computers to perform critical tasks such as disease diagnosis and decision-making by recognising complex patterns in new data based on the prior datasets it was trained with. Deep learning is a ML model based on layers of artificial neural networks [11]. Natural language processing enables computers to understand human language and consequently extract relevant information. This in turn enables sophisticated predictions and medical recognition activities [12]. Similarly, AI-based image recognition models have the ability to identify hidden insights in image data, such as micrographs, and medical scans [13]. Expert systems mimic the problem-solving capabilities of human experts; they can solve complex medical problems and serve as an education platform for medical professionals [14].

Some studies have assessed the views of various groups of healthcare professionals regarding the utilisation of AI and its models in healthcare. For instance, a study was undertaken in Australia and New Zealand among clinicians, and AI was identified as having the potential to improve disease screening and streamline repetitive tasks [15]. A similar survey was also carried out among physicians from 54 developed countries, where the cohort reported positive attitudes toward the use of AI for diagnostics [16]. Botwe *et al.* [17]. also assessed African radiographers’ views regarding the use of AI in medical imaging practice, and a majority of the respondents believed that AI technology can improve quality assurance in radiography practice and enhance clinical care.

In addition to the studies highlighted above, other studies have assessed the benefits and applications of AI in different healthcare settings [18–20]. A recurring theme of these surveys was that they were tailored to specific healthcare professional groups rather than a general perspective. While perceptions in developed countries have been studied, a multi-professional, national-level assessment across the full spectrum of AI applications is lacking in Nigeria, which is essential for a holistic national strategy. It is against this backdrop that this study undertook a comprehensive evaluation of health professionals’ views on the applications of AI and ML in the Nigerian healthcare system. The findings from this research will contribute to informed decision-making, policy formulation, and the development of strategies for the effective integration of AI and ML technologies in the Nigerian healthcare sector.

## Methods

### Ethics statement

The study was approved by the National Institute for Pharmaceutical Research and Development Health Research Ethics Committee (Approval number: NIPRD-HREC 18/04/2023-20) prior to the commencement of data collection. Participation in this study was voluntary. Written informed consent was obtained from the participants prior to the administration of questionnaires, and only those who provided consent to partake in the study were issued questionnaires. The information provided was anonymised to ensure confidentiality.

### Study design

The study was undertaken between March and August 2023. This study was carried out using a cross-sectional study design, and responses were obtained from healthcare professionals, including physicians, pharmacists, nurses, medical laboratory scientists, physiotherapists, radiographers, paramedics, and community health extension workers.

### Research Instrument

A comprehensive review of literature was carried out to develop the data collection instrument [15,17,19,20]. The questionnaire (S1 Questionnaire) was designed in the English language. The items in the instrument were divided into eight sections, which include demographic characteristics, questions on application of AI models in research and development, and use of AI algorithms in drug discovery and development. Others are AI technologies in pharmaceutical manufacturing, applications of AI innovations in clinical settings, AI in drug therapy, AI for process optimisation and the applications of AI in public healthcare in Nigeria. A Likert scale was used to gauge responses (1 = Strongly Disagree, 2 = Disagree, 3 = Neutral, 4 = Agree and 5 = Strongly Agree). Face and content validations were undertaken by an expert panel comprising researchers with experience in this area. Construct validity was ensured by grouping items into predefined domains reflecting established themes in the literature. Pilot testing was then carried out by administering the questionnaire to an initial cohort of 20 randomly selected participants, and Cronbach alpha’s test (α = 0.834) was carried out to affirm the reliability of the instrument. The feedback at the conclusion of the pilot testing resulted in no major change to the data collection tool.

### Inclusion and exclusion criteria

The inclusion criteria for participants in the study were healthcare professionals registered to practice in Nigeria, willingness to participate, and with a current annual licence to practice. Participants who did not meet these criteria were excluded from the study.

### Data collection procedure

Data were collected through a multistage sampling technique. First, a state was randomly selected from each of Nigeria’s six geopolitical zones (North Central, North East, North West, South East, South South, and South West). Data were aggregated across all six zones to form a nationally representative sample; the study was not powered for comparative sub-national analysis.

Within each selected state, healthcare facilities were stratified into public and private institutions, and facilities were randomly selected from each stratum. Finally, healthcare professionals meeting the inclusion criteria were invited to participate, with efforts made to include a diverse range of professions and years of practice.

### Sample size

Epi Info software version 7 was used to calculate the minimum sample size required for a population of 0.94 million healthcare workers in Nigeria [21]. This was computed at a 95% confidence level, 5% margin of error, and a 50% response distribution. The calculated sample size was 384; this was however, rounded up to 500 so as to account for non-response.

### Data analysis

Data collected were coded into Statistical Package for Social Sciences (SPSS) version 25 and then analysed. Attitudes of the participants regarding the applications of AI technologies in Nigerian healthcare were assessed with a total of twenty-three (23) statements that utilised a five-point Likert scale to capture participants’ views on relevant thematic areas. Univariate analysis was carried out to yield descriptive statistics, and the results were presented in frequencies and percentages. A chi-square test was undertaken to determine the association between responses and socio-demographic characteristics. A *p*-value of < 0.05 represented the threshold for statistical significance. Only the statistically significant demographic characteristics were reported. A multinomial logistic regression was also carried out to identify predictors of perception toward AI and ML adoption. Missing data were reviewed before analysis to identify incomplete responses across all variables. The overall volume of missing data was low, and items with missing responses were excluded from the analysis of those specific variables only. As indicated in the tables, all percentages were calculated based on valid responses. Missing entries were likely due to participants unintentionally skipping non-mandatory items during questionnaire completion. Given the low and non-systematic nature of the missing data, its impact on the study findings is considered minimal.

## Results

### Demography

Out of 500 questionnaires administered to respondents, a total of 404 were completed and recovered, indicating a response rate of 80.8%. Pharmacists accounted for 27.7% of the participants, followed by physicians (24.5%), and nurses (19.3%). The study recorded a higher rate of female participants (55.7%) compared to males (44.3%). More than half of the participants (68.3%) were within the age range of 18–30 years, whilst participants aged 60 years (1.2%) and above, were least represented. Further details on socio-demographic characteristics are presented in Table 1.

**Table 1:** Socio-demographic characteristics of the respondents (n = 404).

Variable	Frequency (%)
Gender	
Male	179 (44.3)
Female	225 (55.7)
Age	
18-30	276 (68.3)
31-40	84 (20.8)
41-50	26 (6.4)
51-60	13 (3.2)
Above 60	5 (1.2)
Profession	
Physician	99 (24.5)
Pharmacist	112 (27.7)
Nurse	78 (19.3)
Medical Lab Scientist	37 (9.2)
Physiotherapist	26 (6.4)
Others	52 (12.9)
Highest Educational Qualification	
Diploma	23 (5.7)
First degree	332 (82.8)
Master’s degree	41 (10.2)
Doctorate	5 (1.2)
Years of Practice	
Less than 5years	285 (71.3)
5-10years	61 (15.3)
11-15years	25 (6.3)
Above 15years	29 (7.2)
Sector	
Government Sector	279 (71.7)
Private Sector	102 (26.2)
Others	8 (2.1)

Note: Missing data were not included in the final percentages presented.

### Applications of AI and ML in Nigerian healthcare

**Fig 1:**
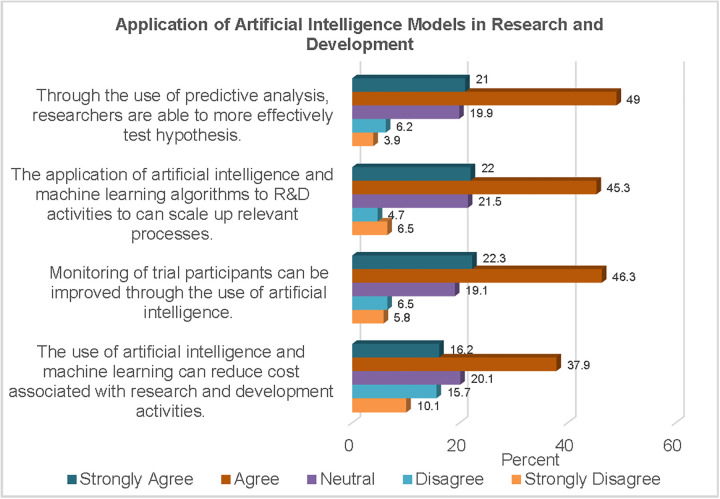
Application of AI Models in Research and Development.

The responses to statements regarding the utilisation of AI in research and development are presented in Fig 1. More than two-thirds of the participants (70%) agreed that AI was more efficient in hypothesis testing, especially through the deployment of predictive analysis capabilities.

At least two-thirds of the respondents (67.3%) believed that ML algorithms could scale up relevant processes in research. Furthermore, more than half of the study sample (54.1%) were of the opinion that AI had the ability to reduce costs associated with research and development activities.

In the area of drug discovery, more than two-thirds of the respondents (69.5%) believed that AI could expedite the process. Similarly, three-quarters of the respondents (75.5%) believed that AI could speed up pharmaceutical product development.

**Fig 2:**
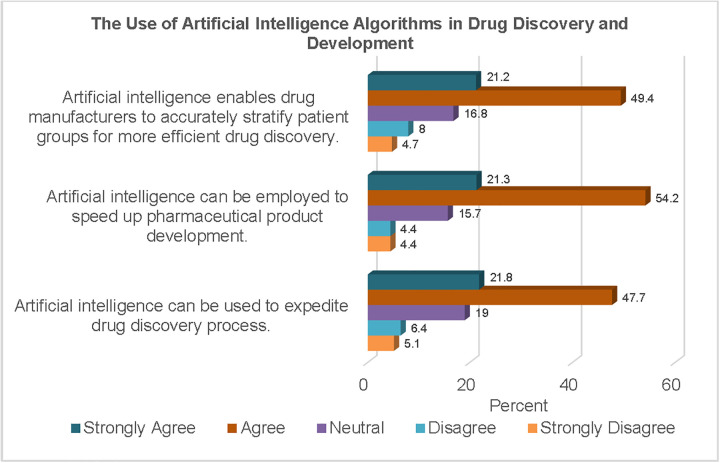
The Use of AI Algorithms in Drug Discovery and Development.

Fig 2 shows the frequencies of responses to statements regarding the use of AI algorithms in the processes involved in drug discovery and development. Close to three-quarters of the study sample (70.6%) agreed that AI could be employed by drug manufacturers to stratify patient groups for more efficient drug discovery.

**Fig 3:**
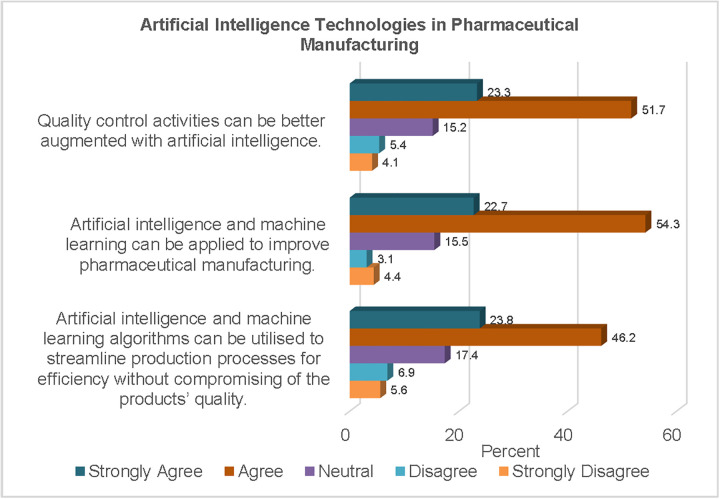
AI Technologies in Pharmaceutical Manufacturing.

As shown in Fig 3, a majority of the participants (77%) agreed that AI and ML could be applied to improve pharmaceutical manufacturing. Whilst a similar proportion of the respondents (75%) believed that quality control as an aspect of pharmaceutical manufacturing could be better augmented with AI technologies.

More than two-thirds of the respondents (70%) agreed that AI and ML algorithms could improve production process efficiencies whilst maintaining relevant quality standards.

**Fig 4:**
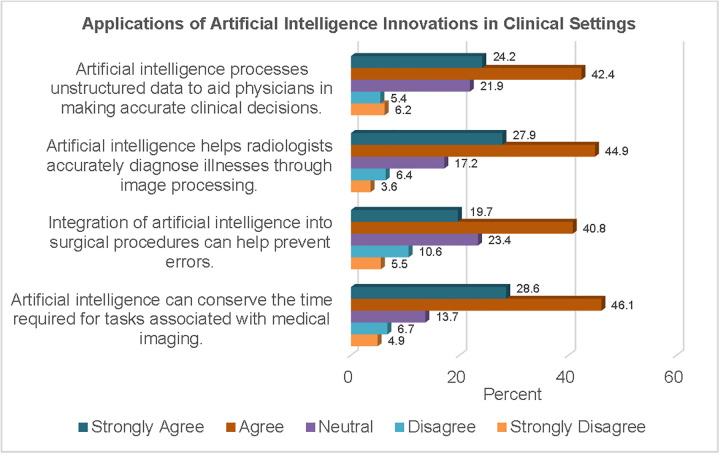
Applications of AI Innovations in Clinical Settings.

Fig 4 shows the percentages of responses concerning the application of AI in different aspects of clinical settings. Regarding decision-making, about two-thirds of the study sample (66.6%) agreed that AI can aid physicians in making accurate clinical decisions through the processing of unstructured data.

Also, more than half of the respondents (60.5%) were of the opinion that the integration of AI into surgical procedures could help prevent errors. In the area of medical imaging, more than half of the participants agreed that AI can conserve time (74.7%) and assist radiologists in making accurate diagnoses through image processing (66.6%).

### Benefits of AI and ML in Nigerian Healthcare

Regarding the benefits in drug therapy, two-thirds of the study sample (68.1%) agreed that AI can be employed in the prediction of drug interactions. In the same vein, more than half of the participants (59.2%) believed that the incidents of adverse drug reactions can be reduced with the adoption of ML models.

**Fig 5:**
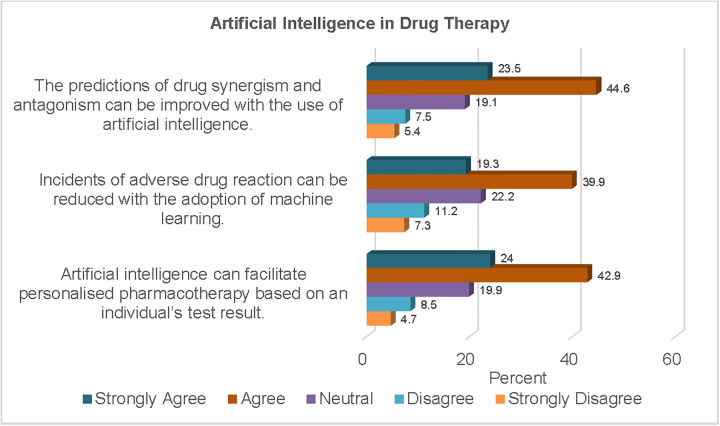
AI in Drug Therapy.

Other information regarding responses to the use of AI in drug therapy are presented in Fig 5. At least two-thirds of the study sample (66.9%) believed that AI could facilitate personalised pharmacotherapy based on patients’ test results.

**Fig 6:**
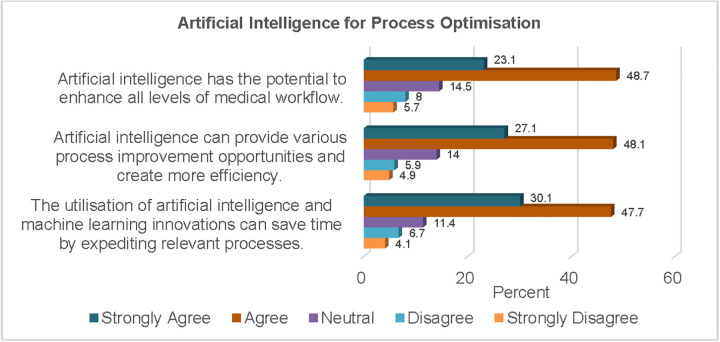
AI for Process Optimisation.

Fig 6 shows the responses of the study sample regarding the utilisation of AI for process optimisation in healthcare where more than two-thirds (71.8%) of the respondents agreed that it had the potential to enhance all levels of medical workflow.

With respect to process improvement, three-quarters of the respondents (75.2%) believed that AI technologies catalysed innovation and enabled process efficiencies. More than three-quarters of the study sample (77.8%) were of the opinion that AI innovations can save time thereby expediting relevant processes.

**Fig 7:**
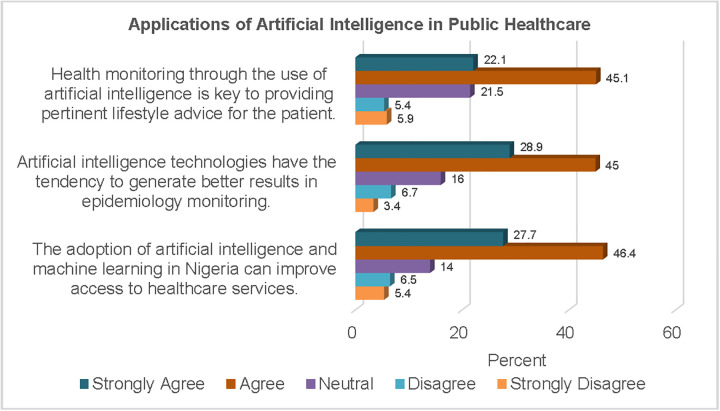
Applications of AI in Public Healthcare.

In public health, two-thirds of the participants (67.2%) concurred that the use of AI in health monitoring is key to the provision of pertinent lifestyle advice for patients. Fig 7 shows the percentage response to statements regarding the application of AI for process optimisation.

Additionally, close to three-quarters of the study sample (73.9%) agreed that AI could generate better results in epidemiology monitoring. A similar proportion of the respondents (74.1%) also believed that the adoption of AI and ML could improve access to healthcare services in Nigeria.

### Association between demography and views of participants

The cross-tabulation between the profession and the perception regarding the application of AI in drug discovery was undertaken. Findings that emerged show more physicians were in agreement with the use of AI to expedite drug discovery as compared to other professionals (*p* = 0.008). Details are presented in Table 2.

**Table 2:** Association between Profession and Views Regarding the Application of AI Models in Drug Discovery.

Statement	Profession	Strongly Disagree (%)	Disagree (%)	Neutral (%)	Agree (%)	Strongly Agree (%)	X^2^	*p-*value
AI can be used to expedite drug discovery process.	Physician	4(4.2)	1(1.0)	15(15.6)	51(53.1)	25(26.0)	38.494	0.008
	Pharmacist	5(4.8)	6(5.7)	18(17.1)	41(39.0)	35(33.3)		
	Nurse	5(6.4)	7(9.0)	17(21.8)	35(44.9)	14(17.9)		
	Medical Laboratory Scientist	2(5.7)	4(11.4)	10(28.6)	14(40.0)	5(14.3)		
	Physiotherapist	0(0.0)	1(3.8)	8(30.8)	16(61.5)	1(3.8)		
	Others	4(8.0)	6(12.0)	6(12.0)	29(58.0)	5(10.0)		

The results of the inferential analysis of the highest educational qualification of participants and their views regarding the application of AI algorithms in research and development shows that more of the doctorate degree holders supported this concept (*p* = 0.036). Further details are presented in Table 3.

**Table 3:** Association between Highest Educational Qualification and Views Regarding the Applications of AI Models.

Statement	Educational Qualification	Strongly Disagree (%)	Disagree (%)	Neutral (%)	Agree (%)	Strongly Agree (%)	X^2^	*p-*value
The use of AI and ML can reduce cost associated with research and development activities.	Diploma	4(17.4)	4(17.4)	3(13.0)	12(52.2)	0(0.0)	22.142	0.036
	First degree	33(10.3)	51(16.0)	70(21.9)	117(36.7)	48(15.0)		
	Master’s degree	2(5.1)	5(12.8)	4(10.3)	15(38.5)	13(33.3)		
	Doctorate	0(0.0)	1(25.0)	0(0.0)	1(25.0)	2(50.0)		

Apart from profession and highest educational qualification, no other socio-demographic variables (gender, age, years of practice, or workplace sector) showed a statistically significant association with the perception items at p < 0.05.

### Multinomial logistic regression analysis

Multinomial logistic regression examined predictors of perception toward AI and ML adoption, with positive perception as the reference category. The model showed acceptable fit (Pearson χ² = 188.20, p = .251; Deviance χ² = 175.69, p = .492) and modest explanatory power (Nagelkerke R² = .068).

Likelihood ratio tests indicated that gender (χ² = 7.13, p = .028) and workplace sector (χ² = 6.56, p = .038) significantly contributed to the model.

### Negative vs Positive Perception

Respondents in the private sector were significantly less likely to report negative perceptions compared with those in the public sector (β = −1.195, OR = 0.30, 95% CI: 0.09–0.97, p = .045), representing a 70% reduction in odds. Gender and other variables were not significant.

### Neutral vs positive perception

Female respondents were more likely than males to report neutral rather than positive perceptions (β = 0.573, OR = 1.77, 95% CI: 1.13–2.78, p = .012), indicating a 77% increase in odds. Workplace sector and other variables were not significant.

## Discussion

A number of insightful findings emerged, indicating professional confidence in the ability of AI and ML to strengthen health research and development processes. Participants recognised that predictive analytics can enhance hypothesis testing by helping researchers determine whether their hypotheses are supported or contradicted by existing data [22]. A major drive for undertaking research is the availability of resources and funding. More than half of the respondents believed that the adoption of AI and ML technologies in the health sector would result in the reduction of costs associated with research and development activities. This aligns with previous findings, which reported similar cost-saving potential [23,24]. This cost reduction is possible through optimised resource allocation, improved decision-making, and fewer errors [25]. Furthermore, two-thirds of the health professionals recruited for this study agreed that AI may be applied in the monitoring of trial participants. This finding is consistent with the work of Olawade *et al.*, who also noted the potential of AI to support clinical trial oversight [26]. ML models can track patients in real time, detect non-compliance, and promote appropriate interventions [27]. These findings on the applications of AI in health research, are consistent with previous reports [28,29].

Participants expressed strong support for AI-driven acceleration of drug discovery and pharmaceutical product development. ML algorithms may be applied to analyse vast amounts of biological data such as genetic information, chemical data, and clinical data to identify potential drug targets [30]. The perceived value of AI for patient stratification further suggests recognition of its role in enhancing trial design and precision medicine. These positive attitudes toward integrating AI in drug discovery and development may be explained by the wide range of algorithmic models available for developing drug candidates and screening lead compounds [31–35].

Three-quarters of the study sample agreed that pharmaceutical manufacturing processes can be improved by AI and ML. AI driven technologies such as process optimisation algorithms can enhance manufacturing efficiency by improving production and reducing costs. Professionals highlighted enhancing quality and streamlining processes as additional benefits. Other studies have also documented the applications of ML in various aspects of pharmaceutical manufacturing [36–38].

Close to three-quarters of the respondents agreed that AI could reduce the time needed for tasks associated with medical imaging. Similarly, majority of the respondents concurred that AI image processing can help radiologists diagnose illnesses more accurately, and Coppola *et al.* [39] reported similar views. Aside from medical imaging, a substantial proportion of the respondents believed that AI could aid physicians in making accurate clinical decisions by processing unstructured data, and these findings are comparable with extant literature [16,40].

Regarding the benefits of AI in healthcare, approximately two-thirds of the study sample believed that it could facilitate personalised pharmacotherapy. AI can facilitate personalised pharmacotherapy by analysing the patient’s unique clinical information and real-time monitoring data. This would enable the algorithm to provide evidence-based recommendations to inform treatment decisions. The use of multiple drugs concurrently may result in interactions between the active compounds. Although some of those interactions may be beneficial, others may result in therapeutic failure or adverse effects. Two-thirds of the study sample agreed that AI may be used to predict drug synergism and antagonism. More than half of the study sample identified ML as an invaluable tool for reducing the incidence of adverse drug reactions. These perceptions align with findings from other studies [41,42].

More than two-thirds of the study sample were of the opinion that AI has the potential to enhance all levels of medical workflow. In Nigerian hospitals, the complexities of seeking medical care can be arduous, potentially dissuading individuals from achieving optimal resolution for interventions sought [43]. An obvious consequence of this is the worsening of health problems, which may contribute to increased mortality. This finding thus highlights the potential of AI driven innovations to mitigate these challenges, ultimately contributing to more effective and accessible healthcare in Nigeria. Nearly three-quarters of the participants also held the viewpoint that the capacity of AI to accelerate processes could be harnessed to save time and provide opportunities for process improvement. Letourneau-Guillon *et al.* [44]. also highlighted different areas of clinical practice that could be automated with the aid of AI innovations.

Furthermore, findings from this study show that the majority of the respondents believed that AI and ML can improve healthcare access in Nigeria. AI-powered telemedicine could facilitate the extension of medical expertise to underserved areas through remote consultations, diagnosis, and health promotion. In addition, automation of medical workflow would enhance the productivity of health providers. Close to three-quarters of the respondents agreed that AI technologies have the capability to generate better results in epidemiological monitoring. By monitoring disease prevalence, environmental factors, and population behaviours, AI could provide forecasts of disease outbreaks and spread in specific areas. More than two-thirds of the study participants agreed that AI-based health monitoring was essential for providing relevant lifestyle advice for patients. Insights generated from AI-based health monitoring in the population can facilitate targeted health promotion campaigns necessary to reduce the risk of specific conditions in different population groups.

A cross-tabulation of respondents’ demographic characteristics and views revealed that physicians displayed the highest level of agreement regarding the use of AI to accelerate the process of drug discovery. Medical doctors are frontline health providers who are continually faced with challenges during diagnosis, treatment, and other processes in the provision of healthcare to the public. Hence, it is plausible that they recognise how AI algorithms can be applied to aid these processes. Doctorate degree and master’s degree holders showed the strongest agreement with the statement regarding the potential of AI to reduce costs associated with research and development. Advanced degree holders typically possess an in-depth knowledge of research methodologies; hence, they are more likely to recognise how AI can optimise resource allocation, increase productivity, as well as reduce time and costs.

To further investigate which demographic and professional factors significantly influence perception, a multinomial logistic regression was conducted. Gender and workplace sector emerged as the only significant predictors of perception of AI and ML, while age, profession, education level, and years of practice were not. The significant associations between gender and perception may reflect underlying differences in access to digital skills, technology use, and digital literacy among healthcare professionals. Studies on health information technology adoption have documented how systemic gender disparities, including fewer opportunities for technical training and sociocultural norms that constrain women’s engagement with digital tools, can influence confidence and attitudes toward technology use among female professionals [45]. Similarly, workplace sector effects likely reflect differences in organisational readiness and infrastructure. Private healthcare facilities in Nigeria are often better resourced with digital infrastructure and more flexible training opportunities, whereas public sector environments frequently face resource limitations, weaker institutional support, and infrastructural barriers that can temper positive perceptions [46]. These dynamics align with broader evidence showing that training, organisational support, and facilitating conditions are important determinants of healthcare workers’ attitudes towards health technology adoption [47].

These results highlight that while healthcare professionals in Nigeria are optimistic about AI applications, actual adoption depends on contextual factors, including gender, organisational context, and access to resources. Practical deployments in low- and middle-income countries (LMICs), such as student health monitoring systems in Indonesia, have shown positive impacts on awareness and lifestyle changes among medical trainees [48].

Emerging evidence from other LMICs indicates that healthcare professionals similarly express strong optimism toward AI applications in diagnostics, workflow efficiency, and improved access, despite limited prior exposure [49–52]. However, implementation remains limited across LMICs due to algorithmic biases from models trained in high-income countries, as well as other factors such as infrastructural deficits, poor local data quality, funding shortages, and regulatory gaps. [53–56]. Nigeria mirrors these broader trends, underscoring that positive perceptions alone are insufficient; equitable large-scale adoption will require investment in digital infrastructure, workforce training, locally relevant datasets, and governance tailored to local contexts.

## Limitations

Although this study offers valuable insights into Nigerian healthcare professionals’ perceptions of AI and ML, it has several limitations. The sample over-represents younger professionals (68.3% aged 18–30 years) and may not fully represent older practitioners or those in rural and private settings. Reliance on self-reported data introduces the possibility of social desirability bias. Furthermore, most participants likely had limited hands-on experience with functional AI systems in Nigerian facilities, making responses largely hypothetical and potentially influenced by awareness of global AI trends and expectations of solutions to systemic healthcare challenges. As the study measures perception, not actual knowledge, experience, or competency with AI/ML. The study also pays minimal attention to potential barriers such as infrastructure deficits, ethical concerns, job displacement fears, and regulatory challenges that could hinder actual adoption. These limitations suggest that while perceptions are largely favourable, further qualitative and longitudinal research will be invaluable for assessing real-world readiness and implementation bottlenecks.

## Conclusion

This study revealed the perceptions of professionals regarding the applications of AI and ML in different aspects of healthcare. Participants expressed the view that the benefits of AI outweigh the initial cost of implementation, thereby saving costs in the long run. Additionally, they believed that the predictive analysis capabilities of AI models could be valuable for hypothesis testing. AI algorithms were also identified as potentially beneficial in drug discovery and development through their ability to expedite relevant processes and save time. Quality control mechanisms, augmentation, and streamlining of production processes were among the benefits identified by the study sample in pharmaceutical manufacturing. Furthermore, process optimisation, improved health access, enhanced epidemiology monitoring and health promotion were also identified as potential benefits of AI implementation in the Nigerian health sector.

These findings indicate the readiness of the health workforce for large-scale implementation of AI technologies in the health sector.

Despite the positive views expressed regarding the applications of AI and ML in the health sector, it is important to note that the actual presence of AI technologies in Nigeria remains quite limited. This reflects persistent gaps in digital infrastructure, local data availability, workforce training, and governance frameworks. Bridging the gap in this area will require coordinated, multi-stakeholder and parallel actions, including targeted investment in digital and data infrastructure. It is also important to improve the development of locally relevant and well-governed health data repositories. Furthermore, the integration of tailored AI and ML training into healthcare education and professional development, as well as the establishment of robust ethical, legal, and regulatory guidelines is critical. Without these complementary structural and policy interventions, positive attitudes toward AI may not translate into meaningful health system transformation in Nigeria. Further studies are recommended to assess various areas where AI technologies have been implemented in Nigeria, to identify relevant gaps that need to be addressed to improve subsequent deployment.

## Supporting information

S1 Questionnaire Prospects of Artificial Intelligence and Machine Learning Innovations from the Perspectives of Nigerian Healthcare Professionals. (DOCX)

S1 Dataset Dataset. (SAV)

## References

[1] Ashley EA. Towards precision medicine. Nat Rev Genet. 2016;17(9):507–22. doi: 10.1038/nrg.2016.86 27528417

[2] Muhammad F, Abdulkareem JH, Chowdhury AA. Major public health problems in Nigeria: A review. SE Asia J Pub Health. 2017;7(1):6–11. doi: 10.3329/seajph.v7i1.34672

[3] Reddy S, Fox J, Purohit MP. Artificial intelligence-enabled healthcare delivery. J R Soc Med. 2019;112(1):22–8. doi: 10.1177/0141076818815510 30507284 PMC6348559

[4] Johnson KB, Wei WQ, Weeraratne D, et al. Precision Medicine, AI, and the Future of Personalized Health Care. Clin Transl Sci. 2021;14(1):86–93. doi: 10.1111/cts.12884 32961010 PMC7877825

[5] Richens JG, Lee CM, Johri S. Improving the accuracy of medical diagnosis with causal machine learning. Nat Commun. 2020;11(1):3923. doi: 10.1038/s41467-020-17419-7 32782264 PMC7419549

[6] Bhaskar S, Bradley S, Sakhamuri S. Designing Futuristic Telemedicine Using Artificial Intelligence and Robotics in the COVID-19 Era. Front Public Health. 2020;8:556789. doi: 10.3389/fpubh.2020.556789 33224912 PMC7667043

[7] Hamet P, Tremblay J. Artificial intelligence in medicine. Metabolism. 2017;69S:S36–40. doi: 10.1016/j.metabol.2017.01.011 28126242

[8] Tavakoli M, Carriere J, Torabi A. Robotics, smart wearable technologies, and autonomous intelligent systems for healthcare during the COVID‐19 pandemic: An analysis of the state of the art and future vision. Adv Intell Syst. 2020;2(7):2000071. doi: 10.1002/aisy.202000071

[9] Amisha MP, Pathania M, Rathaur VK. Overview of artificial intelligence in medicine. J Family Med Prim Care. 2019;8(7):2328–31. doi: 10.4103/jfmpc.jfmpc_440_19 31463251 PMC6691444

[10] Ganasegeran K, Abdulrahman SA. Artificial Intelligence Applications in Tracking Health Behaviors During Disease Epidemics. Learning and Analytics in Intelligent Systems. Springer International Publishing. 2019. p. 141–55. doi: 10.1007/978-3-030-35139-7_7

[11] Janiesch C, Zschech P, Heinrich K. Machine learning and deep learning. Electron Markets. 2021;31(3):685–95. doi: 10.1007/s12525-021-00475-2

[12] Hirschberg J, Manning CD. Advances in natural language processing. Science. 2015;349(6245):261–6. doi: 10.1126/science.aaa8685 26185244

[13] Tian Y. Artificial Intelligence Image Recognition Method Based on Convolutional Neural Network Algorithm. IEEE Access. 2020;8:125731–44. doi: 10.1109/access.2020.3006097

[14] Salem H, Soria D, Lund JN, Awwad A. A systematic review of the applications of Expert Systems (ES) and machine learning (ML) in clinical urology. BMC Med Inform Decis Mak. 2021;21(1):223. doi: 10.1186/s12911-021-01585-9 34294092 PMC8299670

[15] Scheetz J, Rothschild P, McGuinness M, Hadoux X, Soyer HP, Janda M, et al. A survey of clinicians on the use of artificial intelligence in ophthalmology, dermatology, radiology and radiation oncology. Sci Rep. 2021;11(1):5193. doi: 10.1038/s41598-021-84698-5 33664367 PMC7933437

[16] Sarwar S, Dent A, Faust K, Richer M, Djuric U, Van Ommeren R, et al. Physician perspectives on integration of artificial intelligence into diagnostic pathology. NPJ Digit Med. 2019;2:28. doi: 10.1038/s41746-019-0106-0 31304375 PMC6550202

[17] Botwe BO, Akudjedu TN, Antwi WK, Rockson P, Mkoloma SS, Balogun EO, et al. The integration of artificial intelligence in medical imaging practice: Perspectives of African radiographers. Radiography (Lond). 2021;27(3):861–6. doi: 10.1016/j.radi.2021.01.008 33622574

[18] Rehm J, Shield KD. Global Burden of Disease and the Impact of Mental and Addictive Disorders. Curr Psychiatry Rep. 2019;21(2):10. doi: 10.1007/s11920-019-0997-0 30729322

[19] Liyanage H, Liaw ST, Jonnagaddala J. Artificial Intelligence in Primary Health Care: Perceptions, Issues, and Challenges. Yearb Med Inform. 2019;28(1):41–6. doi: 10.1055/s-0039-1677901 31022751 PMC6697547

[20] Doraiswamy PM, Blease C, Bodner K. Artificial intelligence and the future of psychiatry: Insights from a global physician survey. Artif Intell Med. 2020;102:101753. doi: 10.1016/j.artmed.2019.101753 31980092

[21] World Health Organization (WHO). The State of the Health Workforce in the WHO Africa Region. Geneva: WHO. 2021. https://apps.who.int/iris/bitstream/handle/10665/348855/9789290234555-eng.pdf

[22] Vora LK, Gholap AD, Jetha K, Thakur RRS, Solanki HK, Chavda VP. Artificial Intelligence in Pharmaceutical Technology and Drug Delivery Design. Pharmaceutics. 2023;15(7):1916. doi: 10.3390/pharmaceutics15071916 37514102 PMC10385763

[23] Rakočević T, Markovic M. Assessing the Impact of AI: The Case of the Pharmaceutical Industry. EJBMR. 2024;9(5):70–5. doi: 10.24018/ejbmr.2024.9.5.2461

[24] Saarthee. AI-Driven Approach to Reducing Clinical Trial Costs and Accelerating Time-to-Market - Leading Data & Analytics Services Company. https://saarthee.ai/ai-driven-approach-to-reducing-clinical-trial-costs-and-accelerating-time-to-market/ 2025. 2025 November 18.

[25] Gomez Rossi J, Feldberg B, Krois J, Schwendicke F. Evaluation of the clinical, technical, and financial aspects of cost-effectiveness analysis of artificial intelligence in medicine: scoping review and framework of analysis. JMIR Med Inform. 2022;10(8):e33703. doi: 10.2196/33703 PMC9419048 35969458

[26] Olawade DB, Fidelis SC, Marinze S, Egbon E, Osunmakinde A, Osborne A. Artificial intelligence in clinical trials: A comprehensive review of opportunities, challenges, and future directions. Int J Med Inform. 2026;206:106141. doi: 10.1016/j.ijmedinf.2025.106141 41075423

[27] Motwani A, Shukla PK, Pawar M. Novel framework based on deep learning and cloud analytics for smart patient monitoring and recommendation (SPMR). J Ambient Intell Human Comput. 2021;14(5):5565–80. doi: 10.1007/s12652-020-02790-6

[28] Laï M-C, Brian M, Mamzer M-F. Perceptions of artificial intelligence in healthcare: findings from a qualitative survey study among actors in France. J Transl Med. 2020;18(1):14. doi: 10.1186/s12967-019-02204-y 31918710 PMC6953249

[29] Teng M, Singla R, Yau O. Health Care Students’ Perspectives on Artificial Intelligence: Countrywide Survey in Canada. JMIR Medical Education. 2022;8(1):e33390. doi: 10.2196/33390 PMC8845000 35099397

[30] Paul D, Sanap G, Shenoy S, Kalyane D, Kalia K, Tekade RK. Artificial intelligence in drug discovery and development. Drug Discov Today. 2021;26(1):80–93. doi: 10.1016/j.drudis.2020.10.010 33099022 PMC7577280

[31] Stokes JM, Yang K, Swanson K, Jin W, Cubillos-Ruiz A, Donghia NM, et al. A Deep Learning Approach to Antibiotic Discovery. Cell. 2020;181(2):475–83. doi: 10.1016/j.cell.2020.04.001 32302574

[32] Bung N, Krishnan SR, Bulusu G, Roy A. De novo design of new chemical entities for SARS-CoV-2 using artificial intelligence. Future Med Chem. 2021;13(6):575–85. doi: 10.4155/fmc-2020-0262 33590764 PMC7888348

[33] Zhu J, Wang J, Wang X, Gao M, Guo B, Gao M, et al. Prediction of drug efficacy from transcriptional profiles with deep learning. Nat Biotechnol. 2021;39(11):1444–52. doi: 10.1038/s41587-021-00946-z 34140681

[34] Dhamodharan G, Mohan CG. Machine learning models for predicting the activity of AChE and BACE1 dual inhibitors for the treatment of Alzheimer’s disease. Mol Divers. 2022;26(3):1501–17. doi: 10.1007/s11030-021-10282-8 34327619

[35] Blanco-González A, Cabezón A, Seco-González A, Conde-Torres D, Antelo-Riveiro P, Piñeiro Á, et al. The Role of AI in Drug Discovery: Challenges, Opportunities, and Strategies. Pharmaceuticals (Basel). 2023;16(6):891. doi: 10.3390/ph16060891 37375838 PMC10302890

[36] Doerr FJS, Florence AJ. A micro-XRT image analysis and machine learning methodology for the characterisation of multi-particulate capsule formulations. Int J Pharm X. 2020;2:100041. doi: 10.1016/j.ijpx.2020.100041 32025658 PMC6997304

[37] Jiang J, Peng H-H, Yang Z, Ma X, Sahakijpijarn S, Moon C, et al. The applications of Machine learning (ML) in designing dry powder for inhalation by using thin-film-freezing technology. Int J Pharm. 2022;626:122179. doi: 10.1016/j.ijpharm.2022.122179 36084876

[38] Mészáros LA, Farkas A, Madarász L, Bicsár R, Galata DL, Nagy B, et al. UV/VIS imaging-based PAT tool for drug particle size inspection in intact tablets supported by pattern recognition neural networks. Int J Pharm. 2022;620:121773. doi: 10.1016/j.ijpharm.2022.121773 35487400

[39] Coppola F, Faggioni L, Regge D, Giovagnoni A, Golfieri R, Bibbolino C, et al. Artificial intelligence: radiologists’ expectations and opinions gleaned from a nationwide online survey. Radiol Med. 2021;126(1):63–71. doi: 10.1007/s11547-020-01205-y 32350797

[40] Emani S, Rui A, Rocha HAL, Rizvi RF, Juaçaba SF, Jackson GP, et al. Physicians’ Perceptions of and Satisfaction With Artificial Intelligence in Cancer Treatment: A Clinical Decision Support System Experience and Implications for Low-Middle-Income Countries. JMIR Cancer. 2022;8(2):e31461. doi: 10.2196/31461 35389353 PMC9030908

[41] Aldughayfiq B, Sampalli S. Patients’, pharmacists’, and prescribers’ attitude toward using blockchain and machine learning in a proposed ePrescription system: online survey. JAMIA Open. 2022;5(1):ooab115. doi: 10.1093/jamiaopen/ooab115 35028528 PMC8752039

[42] Liu X, Barreto EF, Dong Y, Liu C, Gao X, Tootooni MS, et al. Discrepancy between perceptions and acceptance of clinical decision support Systems: implementation of artificial intelligence for vancomycin dosing. BMC Med Inform Decis Mak. 2023;23(1):157. doi: 10.1186/s12911-023-02254-9 37568134 PMC10416522

[43] Amedari M, Ejidike II. Improving access, quality and efficiency in health care delivery in Nigeria: a perspective. PAMJ – One Health. 2021;5:3. doi: 10.11604/pamj-oh.2021.5.3.28204

[44] Letourneau-Guillon L, Camirand D, Guilbert F, Forghani R. Artificial Intelligence Applications for Workflow, Process Optimization and Predictive Analytics. Neuroimaging Clinics of North America. 2020;30(4):e1–15. doi: 10.1016/j.nic.2020.08.008 33039002

[45] Moulaei K, Moulaei R, Bahaadinbeigy K. Barriers and facilitators of using health information technologies by women: a scoping review. BMC Med Inform Decis Mak. 2023;23(1):176. doi: 10.1186/s12911-023-02280-7 37670281 PMC10478440

[46] Grandville Medical & Laser. The role of private hospitals in strengthening Nigeria’s healthcare system. Grandville Medical & Laser. https://gml.com.ng/the-role-of-private-hospitals-in-strengthening-nigerias-healthcare-system/ 2025. 2026 February 9.

[47] Edo OC, Ang D, Etu E-E, Tenebe I, Edo S, Diekola OA. Why do healthcare workers adopt digital health technologies - A cross-sectional study integrating the TAM and UTAUT model in a developing economy. International Journal of Information Management Data Insights. 2023;3(2):100186. doi: 10.1016/j.jjimei.2023.100186

[48] Pratama R, Suhanda R, Aini Z, Nurjannah N, Geumpana TA. Application of artificial intelligence technology in monitoring students’ health: Preliminary results of Syiah Kuala Integrated Medical Monitoring (SKIMM). Narra J. 2024;4(2):e644. doi: 10.52225/narra.v4i2.644 39280283 PMC11391959

[49] Ahmed Z, Bhinder KK, Tariq A, Tahir MJ, Mehmood Q, Tabassum MS, et al. Knowledge, attitude, and practice of artificial intelligence among doctors and medical students in Pakistan: A cross-sectional online survey. Ann Med Surg (Lond). 2022;76:103493. doi: 10.1016/j.amsu.2022.103493 35308436 PMC8928127

[50] Habib MM, Hussain T, Butt MA. Knowledge, attitudes, and perceptions of healthcare students and professionals regarding artificial intelligence applications in healthcare: a cross-sectional survey from Pakistan. PLOS Digit Health. 2024;3(3):e0000443. doi: 10.1371/journal.pdig.0000443 PMC11086889 38728363

[51] Alaran MA, Lawal SK, Jiya MH, Egya SA, Ahmed MM, Abdulsalam A, et al. Challenges and opportunities of artificial intelligence in African health space. Digit Health. 2025;11:20552076241305915. doi: 10.1177/20552076241305915 39839959 PMC11748156

[52] Adedinsewo DA, Onietan D, Morales-Lara AC, Moideen Sheriff S, Afolabi BB, Kushimo OA, et al. Contextual challenges in implementing artificial intelligence for healthcare in low-resource environments: insights from the SPEC-AI Nigeria trial. Front Cardiovasc Med. 2025;12:1516088. doi: 10.3389/fcvm.2025.1516088 40134980 PMC11932990

[53] Wahl B, Cossy-Gantner A, Germann S. Artificial intelligence (AI) and global health: how can AI contribute to health in resource-poor settings?. npj Digit Med. 2022;5:162. doi: 10.1038/s41746-022-00692-9 30233828 PMC6135465

[54] Mbunge E, Muchemwa B, Batani J. Application of artificial intelligence and machine learning in healthcare: implications for sub-Saharan Africa. Health Serv Insights. 2023;16:11786329231190745. doi: 10.1177/11786329231190745

[55] World Health Organization WHO. Ethics and governance of artificial intelligence for health: guidance on large multi-modal models. Geneva: World Health Organization. 2021. https://www.who.int/publications/i/item/9789240039858

[56] Ciecierski-Holmes T, Wahl B, Otwoma N. Strengthening healthcare systems in low- and middle-income countries with artificial intelligence: scoping review update. npj Digit Med. 2022;5:162. doi: 10.1038/s41746-022-00692-9 36307479 PMC9614192

