# Supplementary material for: Perception of artificial intelligence and machine learning applications in the Nigerian healthcare sector: A cross-sectional study
Source: PLOS Glob Public Health. 2026 May 28;6(5):e0006124. doi: 10.1371/journal.pgph.0006124 (PMC13218525; doi:10.1371/journal.pgph.0006124)
Supplement: S1 Questionnaire — (DOCX) [file pgph.0006124.s001.docx]

**Appendix I: Questionnaire**

**Prospects of Artificial Intelligence and Machine Learning Innovations from the Perspectives of Nigerian Healthcare Professionals**

Artificial intelligence may be employed in different aspects of healthcare including, research and development; clinical decision-making; pharmaceutical manufacturing; and public healthcare. This questionnaire aims to evaluate the prospects of artificial intelligence and machine learning from the focal points of healthcare professional groups in Nigeria. Please fill out the questionnaire by ticking (√) the most appropriate option (s). Your responses will be anonymised and treated confidentially.

**Demographic Data**

1. **Gender**

| Male | Female |
| --- | --- |

1. **Age**

| 18-30 | 31-40 | 41-50 | 51-60 | Above 60 |
| --- | --- | --- | --- | --- |

1. **Profession**

| Physician | Pharmacist | Nurse | Medical Laboratory Scientists | Physiotherapist | Others, please specify.  ……………….. |
| --- | --- | --- | --- | --- | --- |

1. **Highest Educational Qualification**

| Diploma | First degree | Master’s degree | Doctorate |
| --- | --- | --- | --- |

1. **Number of Years of Practice**

| <5years | 5-10years | 11-15years | Above 15years |
| --- | --- | --- | --- |

1. **Sector**

| Government Sector | Private Sector | Others, please specify. ………………………………. |
| --- | --- | --- |

1. **Application of Artificial Intelligence Models in Research and Development**

| **SN** | **Statements** | **Strongly Disagree** | **Disagree** | **Neutral** | **Agree** | **Strongly Agree** |
| --- | --- | --- | --- | --- | --- | --- |
|  | Through the use of predictive analysis, researchers are able to more effectively test hypothesis. |  |  |  |  |  |
|  | The application of artificial intelligence and machine learning algorithms to R&D activities can scale up relevant processes. |  |  |  |  |  |
|  | Monitoring of trial participants can be improved through the use of artificial intelligence. |  |  |  |  |  |
|  | The use of artificial intelligence and machine learning can reduce cost associated with research and development activities. |  |  |  |  |  |

1. **The Use of Artificial Intelligence Algorithms in Drug Discovery and Development**

| **SN** | **Statements** | **Strongly Disagree** | **Disagree** | **Neutral** | **Agree** | **Strongly Agree** |
| --- | --- | --- | --- | --- | --- | --- |
|  | Artificial intelligence enables drug makers to accurately stratify patient groups for more efficient drug discovery. |  |  |  |  |  |
|  | Artificial intelligence can be employed to speed up pharmaceutical product development. |  |  |  |  |  |
|  | Artificial intelligence can be used to expedite drug discovery process. |  |  |  |  |  |

1. **Artificial Intelligence Technologies in Pharmaceutical Manufacturing**

| **SN** | **Statements** | **Strongly Disagree** | **Disagree** | **Neutral** | **Agree** | **Strongly Agree** |
| --- | --- | --- | --- | --- | --- | --- |
|  | Quality control activities can be better augmented with artificial intelligence. |  |  |  |  |  |
|  | Artificial intelligence and machine learning can be applied to improve pharmaceutical manufacturing. |  |  |  |  |  |
|  | Artificial intelligence and machine learning algorithms can be utilised to streamline production processes for efficiency without compromising of the products’ quality. |  |  |  |  |  |

1. **Applications of Artificial Intelligence Innovations in Clinical Settings**

| **SN** | **Statements** | **Strongly Disagree** | **Disagree** | **Neutral** | **Agree** | **Strongly Agree** |
| --- | --- | --- | --- | --- | --- | --- |
|  | Artificial intelligence processes unstructured data to aid physicians in making accurate clinical decisions. |  |  |  |  |  |
|  | Artificial intelligence helps radiologists accurately diagnose illnesses through image processing. |  |  |  |  |  |
|  | Integration of artificial intelligence into surgical procedures can help prevent errors. |  |  |  |  |  |
|  | Artificial intelligence can conserve the time required for tasks associated with medical imaging. |  |  |  |  |  |

1. **Artificial Intelligence in Drug Therapy**

| **SN** | **Statements** | **Strongly Disagree** | **Disagree** | **Neutral** | **Agree** | **Strongly Agree** |
| --- | --- | --- | --- | --- | --- | --- |
|  | The predictions of drug synergism and antagonism can be improved with the use of artificial intelligence. |  |  |  |  |  |
|  | Incidents of adverse drug reaction can be reduced with the adoption of machine learning. |  |  |  |  |  |
|  | Artificial intelligence can facilitate personalised pharmacotherapy based on an individual’s test result. |  |  |  |  |  |

1. **Artificial Intelligence for Process Optimisation**

| **SN** | **Statements** | **Strongly Disagree** | **Disagree** | **Neutral** | **Agree** | **Strongly Agree** |
| --- | --- | --- | --- | --- | --- | --- |
|  | Artificial intelligence has the potential to enhance all levels of medical workflow. |  |  |  |  |  |
|  | Artificial intelligence can provide various process improvement opportunities and create more efficiency. |  |  |  |  |  |
|  | The utilisation of artificial intelligence and machine learning innovations can save time by expediting relevant processes. |  |  |  |  |  |

1. **Applications of Artificial Intelligence in Public Healthcare**

| **SN** | **Statements** | **Strongly Disagree** | **Disagree** | **Neutral** | **Agree** | **Strongly Agree** |
| --- | --- | --- | --- | --- | --- | --- |
|  | Health monitoring through the use of artificial intelligence is key to providing pertinent lifestyle advice for the patient. |  |  |  |  |  |
|  | Artificial intelligence technologies have the tendency to generate better results in epidemiology monitoring. |  |  |  |  |  |
|  | The adoption of artificial intelligence and machine learning in Nigeria can improve access to healthcare services. |  |  |  |  |  |
